# Print-Light-Synthesis for Single-Step Metal Nanoparticle Synthesis and Patterned Electrode Production

**DOI:** 10.3390/nano13131915

**Published:** 2023-06-23

**Authors:** Stefano Gianvittorio, Domenica Tonelli, Andreas Lesch

**Affiliations:** Department of Industrial Chemistry “Toso Montanari”, University of Bologna, Center for Chemical Catalysis—C^3^, Viale del Risorgimento 4, 40136 Bologna, Italy; stefano.gianvittorio@unibo.it (S.G.); domenica.tonelli@unibo.it (D.T.)

**Keywords:** Print-Light-Synthesis, inkjet printing, digital printing, photochemical deposition, nanomaterial synthesis, thin film electrodes, electrochemistry, additive manufacturing

## Abstract

The fabrication of thin-film electrodes, which contain metal nanoparticles and nanostructures for applications in electrochemical sensing as well as energy conversion and storage, is often based on multi-step procedures that include two main passages: (i) the synthesis and purification of nanomaterials and (ii) the fabrication of thin films by coating electrode supports with these nanomaterials. The patterning and miniaturization of thin film electrodes generally require masks or advanced patterning instrumentation. In recent years, various approaches have been presented to integrate the spatially resolved deposition of metal precursor solutions and the rapid conversion of the precursors into metal nanoparticles. To achieve the latter, high intensity light irradiation has, in particular, become suitable as it enables the photochemical, photocatalytical, and photothermal conversion of the precursors during or slightly after the precursor deposition. The conversion of the metal precursors directly on the target substrates can make the use of capping and stabilizing agents obsolete. This review focuses on hybrid platforms that comprise digital metal precursor ink printing and high intensity light irradiation for inducing metal precursor conversions into patterned metal and alloy nanoparticles. The combination of the two methods has recently been named Print-Light-Synthesis by a group of collaborators and is characterized by its sustainability in terms of low material consumption, low material waste, and reduced synthesis steps. It provides high control of precursor loading and light irradiation, both affecting and improving the fabrication of thin film electrodes.

## 1. Introduction

Nanomaterials, such as metal, alloy, and metal oxide nanoparticles, find wide applications as active materials in electrochemical systems, including electroanalytical sensors and energy storage as well as energy conversion devices [1]. This is generally due to the ability of nanomaterials, alone or as composites, to enhance electrode kinetics of electron transfer reactions, provide ion-selectivity for potentiometric sensing, increase the active surface area of electrodes, or store electric charges in batteries. It is general praxis to synthesize nanoparticles using wet chemical methods, spectroscopically characterize them, and then deposit them on insulating or conductive supports to fabricate the target layer of electrochemical devices. Wet chemistry syntheses of nanoparticles in flasks and reactors show the advantage of being operated under precisely controlled reaction conditions, such as reaction volume, temperature, pressure, stirring rate, atmosphere, and time. It is also possible to add reagents at defined time points. This approach is often used to fabricate nanoparticles of pure metals and alloys, specific nanoparticle structures such as core shell particles, and nanoparticles of specific shapes and morphologies. However, the mass and concentration of reactants as well as the volume of solvents can be quite large compared to the amounts needed for the production of thin film electrodes, which could be micrograms per square centimeter. Considering the challenges currently faced in terms of material availability and sustainability, material synthesis approaches that request low amounts of raw materials and noble metal precursors become not only a major interest but also a major need. During the synthesis, the growth of nanoparticles must be restricted in order to obtain a desired small mean particle size with ideally narrow size distribution. Another challenge is related to the irreversible aggregation and/or agglomeration of the nanoparticles that could influence their properties in the final device application. Therefore, stabilizing agents, such as surfactants and polymers, are generally added to the reaction medium to prevent particle aggregation and agglomeration, for instance, by means of electrostatic and steric interactions.

Once nanoparticles have been prepared in a liquid phase, it might be necessary to separate and wash those nanoparticles from the reaction medium. In order to create an electrode or electrode-modifying film made of the as-synthesized nanoparticles, the identification of a proper method for depositing the nanoparticles is required. For this purpose, the nanoparticles can be dispersed in a liquid phase and deposited onto a solid support, which could be conductive (e.g., glassy carbon (GC), platinum, or gold) or insulating (e.g., polymer films or paper). After the deposition of the nanoparticle dispersion, solvents must evaporate and, depending on the application, nanoparticle-stabilizing agents must be removed from the surfaces of the nanoparticles, as they can act as a contaminant in the desired application (e.g., by blocking active surface sites on the nanoparticles reducing electrode kinetics). The removal of organic stabilizing molecules and polymers is often realized by thermal treatments that are carried out in a muffle furnace representing a time consuming post-deposition process step. On plastic substrates, the temperature that can be applied is low, limiting the efficiency of the removal of certain compounds that require higher temperatures than those plastics can resist to.

Purified nanoparticles may lack adequate adhesion to the support and detach immediately when in contact with fluids during electrochemical applications. In electrochemistry laboratories, it is daily praxis to modify electrode surfaces, such as GC or Pt, with nanoparticles through drop-casting, using nanoparticle dispersions with added binders, such as Nafion or polyvinylidene fluoride resins. It must be noted that binders do not always create problems for the application; sometimes they are even desired, as for instance when catalyst layers in fuel cells are being studied. In those cases, Nafion does not only act as a binder but also as an essential ionic conductor.

Another challenge is related to the transfer of small-scale laboratory fabrication processes into industrial scale production lines, as these could require the design of new dispersions and fluids, as not all laboratory methods are scalable for electrode patterning and large number productions. In order to prepare electrodes and electrode coatings, printing technologies are more frequently used, such as roll-to-roll printing, slot-die coating, or spray coating. Several printing technologies can be used for patterning, such as screen-printing and inkjet printing, to create, for instance, miniaturized electrodes with specific geometries, e.g., interdigitated electrodes. In terms of waste reduction, drop-on-demand ink deposition tools, such as inkjet printing, offer great opportunities as materials are only deposited where demanded, in order to create a desired pattern. Ink excess does not remain on a mask, as is the case with screen printing, and does not need to be washed away as in photolithography.

**Figure 1:**
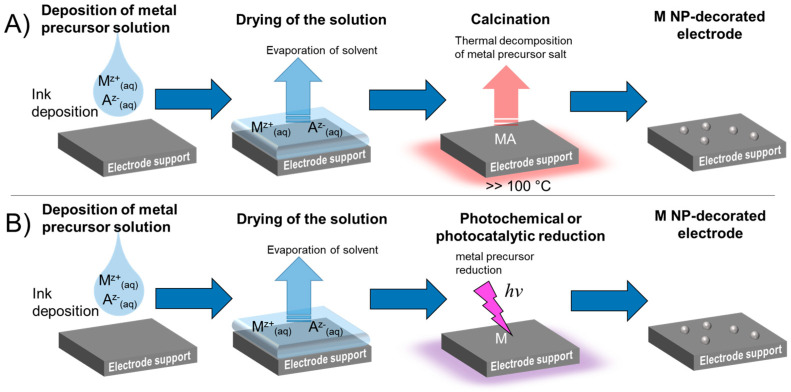
General approaches to creating metal electrode patterns on a large scale based on precursor solutions. (**A**) Deposition of a metal precursor solution and subsequent thermal decomposition of the precursor. Thermal decomposition is an energy- and time-intensive process. (**B**) Deposition of a metal precursor ink with immediate photochemical reduction using high intensity light sources. High intensity flashlamps can reduce the photochemical process time to fractions of a second.

Industrial scale production of electrode patterns combining the printing of metal precursor solutions and the reduction of the metal precursors by thermal processing is an often reported approach, but the latter is time and energy consuming (Figure 1A). Alternatively, high intensity light irradiation, for instance using pulsed irradiation as short as milliseconds, can be used to reduce the metal precursor to the metal with oxidation state zero (Figure 1B) [2]. Using inkjet printing or a similar precursor ink deposition method with high control of film thickness and material loading by the printing parameters, has been called Print-Light-Synthesis by Lesch [3].

Print-Light-Synthesis can be seen as a specialization of reactive inkjet printing, where instead of printing an ink with a reducing agent for the chemical reduction of the previously printed metal precursor, high intensity light irradiation is used to induce the metal precursor reduction. Print-Light-Synthesis differs from photolithographic approaches in the sense that the printing result is controllable not only by the irradiation parameters but also by the printing parameters, such as the controllable precursor loading and film thickness. Furthermore, during Print-Light-Synthesis, all the metal precursor ink that was printed is completely converted, while in photolithographic approaches only irradiated portions are processed and non-irradiated parts have to be removed.

In this review, we presented Print-Light-Synthesis as a method for fabricating patterns of metal nanoparticles for electrode production and modification. We will first briefly present classical approaches to synthesizing nanoparticles using wet chemical methods before depositing them to create thin film nanoparticle patterns and, second, we shall discuss how these two concepts have recently been combined for single step synthesis.

## 2. Fundamentals on Printing and Light-Induced Synthesis of Nanoparticles and Nanoparticle-Decorated Electrodes

### 2.1. Irradition-Free and Irradiation-Induced Nanoparticle Synthesis

Nanoparticles can be synthesized following the bottom-up approach (i.e., the reduction of dissolved precursor metal cations to generate solid matter) or the top-down approach (i.e., the breakdown of larger solid components into nanoparticles). We shall focus herein only on the former. The irradiation-free synthesis of metal nanoparticles from precursor salts can be realized, for instance, by the thermal decomposition of the metal precursor salts on a target support inside a furnace. For noble metals, such as Au, Pt, and Ag, the required temperatures can be relatively mild (i.e., a few hundred degrees Celsius) and the reduction can proceed even in an ambient atmosphere, i.e., in the presence of oxygen [4,5,6]. The feasibility of this process is characterized by the high standard redox potentials of the metal precursor/metal redox couples, such as Au (*E*°_[AuCl_4_]_^−^_/Au_^0^ = 1.00 V), Pt (*E*°_[PtCl_6_]_^4−^_/[PtCl_4_]_^2−^ = 0.68 V, *E*°_[PtCl_4_]_^2−^_/Pt_^0^ = 0.76 V), and Ag (*E*°_Ag^+^/Ag_^0^ = 0.80 V). For these examples, the metal precursors in aqueous solution are, in general, tetrachloroauric acid (HAuCl_4_, dissolved in solution as AuCl_4_^−^), hexachloroplatinic acid (H_2_PtCl_6_, dissolved in solution as PtCl_6_^2−^, and silver nitrate (AgNO_3_, dissolved in solution as Ag^+^). Nitrate is used for silver instead of chloride, as silver halides are hardly soluble.

The reduction of the metal precursors occurs due to electrons that are released from an electron donor, e.g., the salt anions, and accepted by the metal precursor. For less noble metals, such as Fe, in presence of oxygen this reaction can lead to metal oxides M_x_O_y_ and metal oxide-hydroxides M_x_O_y_(OH)_z_ [7]. In oxygen-free environments or reducing atmospheres (e.g., in the presence of hydrogen or carbon monoxide), the thermal reduction of these metal salts can, however, also result in pure metals M^0^. As an alternative, dissolved metal precursors can be chemically reduced in solution by using adequate reducing agents, for instance antioxidants such as ascorbic acid or inorganic species such as borohydride BH_4−_ [8].

Stabilizing and capping agents in wet nanoparticle syntheses are essential for controlling and limiting the growth of the NPs in order to produce the desired shapes and sizes with narrow size distribution. The range of variables in each synthesis is quite wide and many investigations have to be made to obtain the desired nanoparticles [1]. A high ratio of stabilizer to precursor generally favors smaller particle dimensions during the synthesis. Citric acid, for instance, is the most used stabilizer for Au NP synthesis. Common surfactants include sodium dodecyl sulfate (SDS) and cetyl trimetyl ammonium bromide (CTAB), amino acids include histidine and arginine, polymers include alginate, poly vinyl alcohol (PVA) and polyvinyl pyrrolidone (PVP), and polysaccharides include chitosan. In many approaches, the capping agent is also the reducing agent. Another parameter that can influence the size and shape of the nanoparticles is the pH of the solution.

In a third irradiation-free metal synthesis approach that we mention here, metal cations are electrochemically reduced by applying well defined potentials or potential programs to an electrode that provide the electrons for the electrochemical reduction of the metal precursor [9]. The electrons are accepted by the cations near the electrode surface forming metal atoms, clusters and, eventually, nanoparticles and structures that will be deposited on the electrode surface.

The synthesis of metal nanoparticles in solution based on light-irradiation has been applied for over a century [10]. It can be sufficient to let a solution with a noble metal salt rest for hours or days under daylight exposure [11]. This process, however, can be accelerated and controlled using proper irradiation sources. The concept is versatile thanks to the different interactions that can occur between metal precursors and the various energies of photons (i.e., the wavelength) as well as intensities of the radiation emitted by light sources. Typical light sources used for this task emit photons with energies corresponding to visible and UV light or even at shorter wavelengths of the electromagnetic spectrum.

**Figure 2:**
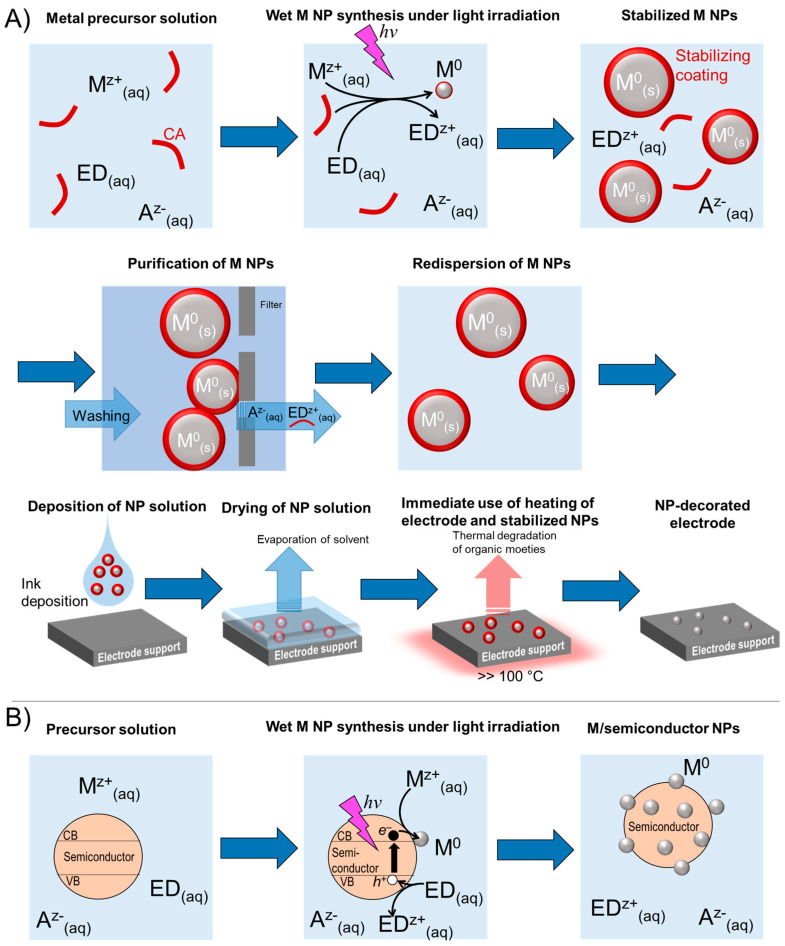
Exemplary routes for electrode patterning of metal nanoparticles that were synthesized by the interaction of electromagnetic radiation with metal precursor cations. (**A**) Schematic representation of photochemical metal nanoparticle synthesis with subsequent thin film fabrication. (**B**) Photocatalytic metal precursor reduction for the synthesis of photocatalytically active metal/semiconductor hybrid nanoparticles.

Major concepts for irradiation-based metal and metal alloy nanoparticle synthesis focus on the photochemical reduction as well as the photocatalytic reduction of metal precursors. The former processes under certain circumstances are driven by the direct interaction of the electromagnetic radiation with the metal precursor or with a photosensitizer inducing the related redox reactions with final metal precursor reduction (Figure 2A). A reaction solution may contain a metal salt (M^z+^-metal cation with positive charge; A^z−^-anion with negative charge z−), an electron donor (ED), and a capping agent (CA). Upon irradiation with proper wavelength and intensity, the metal cation reduces to M^0^ while the electron donor oxidizes to ED^z+^. The nanoparticles are stabilized and protected by the capping agent and purified by washing before a new dispersion is made for subsequent deposition. After deposition, thermal degradation of the NP protection layer is carried out. The photocatalytic process instead uses a light absorbing support material as a photocatalyst (Figure 2B)—in general a semiconductor particle, such as TiO_2_. Under light irradiation with a proper wavelength, an electron-hole pair is generated in the semiconductor nanoparticle, by exciting an electron *e*^−^ from the valence band (VB) to the conduction band (CB), leaving a hole *h*^+^ in the VB, thus reducing the metal precursor and oxidizing an electron donor, such as an alcohol, at the semiconductor surface. The metal is deposited on the semiconductor surface and the M NPs/TiO_2_ composites can be used as photocatalysts in dye-sensitized solar cells and for photocatalytic water splitting.

Many studies on wet metal nanoparticle synthesis have further focused on the control of the size, composition, and morphology of the particles obtained. In the first step, a precursor is reduced to generate single atoms that, eventually, assemble to clusters that act as nuclei for the growth to nanocrystals and nanoparticles. Reducing agents act as electron donors, which are necessary to obtain the metals. Typical examples include, in particular, alcohols such as methanol, ethanol, and isopropanol. Under light irradiation, chemical bonds, such as C-H in the alcohols, vibrate upon excitation and eventually dissociate, forming reactive radicals. The presence of alcohols also scavenges hydroxyl radicals and hydrogen peroxide, which could cause oxidation reactions to counteract with the metal cation reduction. In the top-down approach using light irradiation, for instance when using lasers, bulk materials are photophysically destructed to generate smaller fractions and eventually nanoparticles.

Many light-induced reactions based on conventional white lamps and UV lamps (e.g., LEDs or Xe arc lamps) are slow and, often slower than the slowest printhead translation rate in combined printing-irradiation processes. This results unfortunately in the incomplete reduction of the precursor during printing. Another risk is that the precursor salt precipitates before its conversion, for instance when the solvent evaporation rate is faster than the precursor reduction rate. High intensity light sources, such as lasers (characteristic wavelengths) and high intensity flashlamps (continuous spectrum with high intensity peaks at certain wavelengths), can reduce the process time. Radiolysis of aqueous solutions is reported to dissociate water into the solvated (or hydrated) electron *e*_aq_^−^ and various radicals (e.g., OH) that are strongly oxidizing reagents, thus provoking contrary reactions to metal precursor reduction [12]. Alcohols, such as methanol, can be added to the solution, which scavenge these radicals. The solvated electron, which has an extremely short lifetime of hundreds of nanoseconds, is a strongly reducing species reported to reduce metal cations such as AuCl_4_^−^ to Au^0^. Photolysis caused by nanosecond laser pulses generates excited metal [M*_x_*Cl*_y_*]^z−^ species that disproportionate during various redox processes, generating M^0^, HCl and Cl_2_ [13]. While the direct interaction of, for instance, Au and Pt halides with UV irradiation has been reported, the presence of alcohols facilitates in general metal reduction because alcohols do not only scavenge oxidizing radicals but they also act as efficient reducing agents for the metal cations, as already reported above [14].

Recently, high intensity pulsed Xe flashlamps attracted much attention for advanced additive manufacturing and several systems have been commercialized. The method has been known for decades but recent technological advances enabled the emission of radiation of higher intensities with shorter irradiation periods. The “reinvented” technique is known as photonic curing and, with some technical variations, also as intense pulsed light synthesis and/or photonic flash synthesis [15]. Xe flash lamps emit wavelengths between 300 and 800 nm with durations of a fraction of a second, reaching high energy densities of several J/cm^2^. Irradiating thin patterns of light-absorbing films made of nanoparticles on non-light absorbing substrates, such as plastics that melt at moderate temperatures below 150 °C, including polyethylene terephthalate (PET) or paper, can generate locally in the light-absorbing film temperatures of several hundreds of degrees for extremely short periods. The plastic or paper substrate as a whole remains at a low temperature as the time to equilibrate and thus heat up is insufficient. This enables local curing of the light-absorbing films within microseconds at temperatures that are much higher than the temperatures that non-light absorbing substrates can withstand conventional equilibrium thermal processing, such as in furnaces, over minutes and hours. Only underneath the light-absorbing and heating film the temperature of the plastic support can locally increase, for instance until the melting point, which can improve the adhesion of the light-absorbing film to the plastic substrate [16].

Redox processes simultaneous to thermal curing have been demonstrated, such as the reduction of copper oxide to copper [17]. In addition, these flash lamps can also be used for photochemical and photocatalytic processes, combined or not combined with thermal processes, based on the process conditions and materials used. For instance, Au, Ag, and AuAg nanoparticles have been fabricated in solution using the treatment of the metal precursor salts with a flash lamp [18]. PVP was reported to degrade during the process, forming alcohol radicals that act as reducing agents. In another work, Mo_2_C-based electrocatalyst particles were synthesized on carbon cloth using the flashlight-induced thermal decomposition of a molybdate/graphene precursor [19].

In order to investigate whether a light source emits suitable wavelengths, the emission spectrum of the light source is compared with the absorption spectrum of the ink components. It has frequently been reported that long wavelengths have initiated photochemical processes that require higher energies. In those cases, it was assumed that multiphoton absorption, i.e., the simultaneous absorption of two or more photons of lower energies, led to the electronic excitation of a light-absorbing species [20].

### 2.2. Most Common Printing Techniques Suitable for Print-Light-Synthesis

For Print-Light-Synthesis of electrodes and nanoparticle-coated electrodes, the metal precursor films shall be deposited as stable thin liquid films on a substrate with micrometric lateral resolution. Stable thin liquid films mean that, in the context of Print-Light-Synthesis, the films will remain liquid, containing a completely dissolved precursor rather than a precipitated or crystallized salt. A high control of precursor loading (mass per area), film thickness, and film homogeneity shall further be realized. Material deposition techniques can principally be divided into (a) mask-less and mask-based techniques and (b) into liquid-immersion, spraying, and dropping techniques.

The deposited thin films are processed simultaneously or subsequently with light, heat, or reactive compounds to convert the precursors into the desired solid. In particular, inkjet printing with printheads based on piezo-electric or thermo-resistive actuation has become popular, as it represents a liquid deposition system that employs minimum liquid volumes thanks to its drop-on-demand operation, which ejects droplets on predefined coordinates without masks [21,22]. There are different ways to control the precursor loading on the substrate. First, the precursor concentration in the ink can be controlled. Second, the number of droplets deposited per area can be adjusted. Third, inkjet printing processes can be repeated to linearly increase the precursor loading.

In order to generate stable droplets for inkjet printing, the inks must fulfill certain rheological characteristics, such as surface tension and viscosity. Suitable ranges and relations of these two parameters can be addressed by calculating the dimensionless *Z* number, which, apart from the surface tension *σ* and viscosity *η*, also considers the density of the liquid and a characteristic length, which in general is the diameter of the orifice of the nozzle. In general, a *Z* value between 1 and 10 has been reported to give a good indication about printability using inkjet printing [23]. One very critical point of inkjet printing is the blocking of the nozzles that often occurs when particle-containing inks are used, as too-large particles, particle agglomerates, and particle aggregates can irreversibly block the nozzle orifices. Even though standard printheads contain nozzles with orifices in the range of ~20 μm, particles with diameters larger than 200 nm can create serious printing issues. Therefore, printing nanoparticle-free inks, such as metal precursor solutions for the Print-Light-Synthesis, stabilizes inkjet printing processes significantly.

Another very important aspect of inkjet printing is the interaction of the ink with the substrate surface. Printed single droplets or overlapping droplets that form a pattern shall homogeneously wet the substrate surface but without spreading over too-large areas in order to keep the desired resolution. As a matter of fact, the drying of a printed droplet or pattern creates a flow of material inside the liquid phase, generally towards droplet or pattern extremes, resulting in a ring or frame of accumulated material while the material content in the center is depleted. This phenomenon is known as the coffee ring effect, which can be reduced or avoided by adjusting the ink composition, thermal post-treatment parameters, or surface of the substrate in terms of surface-free energy or porosity.

In almost all cases, when metal precursor films are prepared using inkjet printing, the metal precursor is reduced to the metal by thermal treatments [24]. Another principally useful technique for Print-Light-Synthesis for the controlled deposition of metal precursor ink patterns can be aerosol jet printing [25].

## 3. Print-Light-Synthesis of Electrodes

For the production of two-dimensional patterns of metal nanoparticles, during Print-Light-Synthesis, thin liquid films containing one or more metal precursor are deposited on the target substrate and reduced by the immediate light exposure of the deposited thin liquid film. Importantly, the process must be adjusted in a way that (i) the precursor reduction is at least as fast as printing and (ii) the light intensity is sufficient for highly efficient photo-induced processes. Otherwise, incomplete metal precursor reduction will occur.

**Figure 3:**
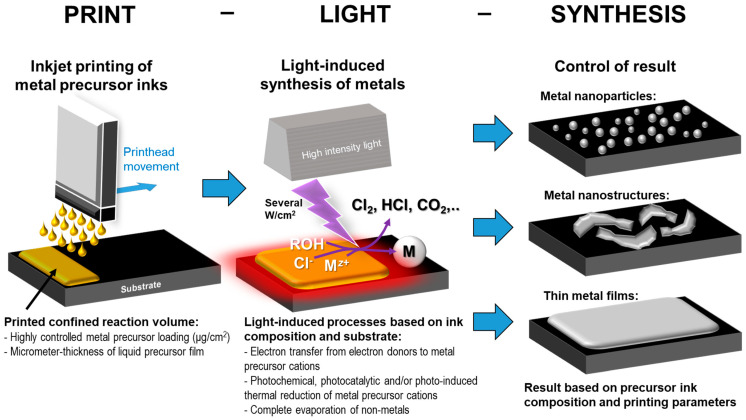
Schematic representation of Print-Light-Synthesis for electrode production. “Print” (left panel): Inkjet printing of a well-defined, confined ultra-thin reaction volume containing one or more dissolved metal precursor salts with highly controllable loading (e.g., few μg/cm^2^). Inkjet printing enables the fabrication of micrometrically resolved patterns. “Light” (central panel): Short irradiation of the as-deposited liquid precursor film converting the metal precursor into the desired material; the vicinity of the precursor to the substrate surface favors the deposition on the substrate rather than the synthesis of particles in the solution. “Synthesis”: The result of the process is a film of metal or mixed metal nanoparticles or nanostructures (depending on the process parameters), while all other ink components have been degraded and/or evaporated.

The thickness of the thin liquid films depends on the amount of ink deposited per area. When using inkjet printing, the thickness of the thin liquid film is controlled by the number of droplets that are deposited per surface area and/or by the droplet size (Figure 3). Moreover, the loading of the metal precursor on the substrate can be precisely controlled by the number of droplets printed per area. Thin liquid film processes, compared to bulk phase processes, are expected to be, on the one hand, more rapid and efficient and, on the other hand, should promote the deposition onto the substrate surface with good adhesion rather than producing loose particles in solution. Therefore, printing thin liquid films is essential for Print-Light-Synthesis. Within the ink, one or more solvents can have a low vapor pressure while the other solvents can quickly evaporate, for instance by heating the substrate to 60 °C. This reduces the ink volume after printing and before light irradiation in a controlled way, leaving very thin liquid films on the substrate. The as-created confined thin liquid reaction volume can, simultaneously or quasi-simultaneously to its formation, be exposed to high intensity light irradiation, such as continuous UV or a flashlight with a pulse sequence as short as milliseconds. The interaction of the thin liquid precursor films with the radiation can initiate light-induced redox reactions (vide supra) and locally heat the thin film, leading to the photochemical, photocatalytical, and/or photothermal synthesis of nanoparticles.

Print-Light-Synthesis is designed in such a way that pure nanomaterials remain on the substrate, while all other components, such as the solvents and other dissolved species, generate gases or evaporate at moderate temperatures. The use of mask-less digital printing techniques provides a large flexibility in terms of pattern design, pattern modification, and process optimization. In the following, various recent examples of Print-Light-Synthesis for electrode production are presented.

### 3.1. Print-Light-Synthesis of Metal Nanoparticles, Nanostructures and Films

**Figure 4:**
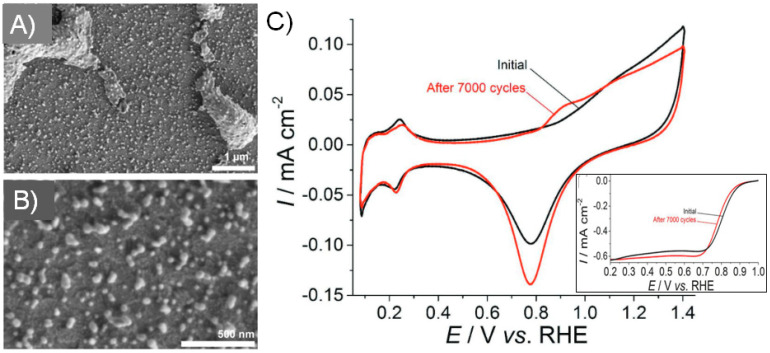
Print-Light-Synthesis of Pt nanoparticles and structures on indium tin oxide-coated glass slides. (**A**,**B**) Scanning electron micrographs demonstrate the presence of Pt nanostructures and/or nanoparticles on the substrate. (**C**) Cyclic voltammograms of a Pt/ITO electrode in nitrogen-saturated 0.1 M HClO_4_ before (black) and after 7000 cycles performed in a separate measurement for oxygen reduction (red). Inset: Linear sweep voltammograms of a Pt/ITO electrode in oxygen-saturated 0.1 M HClO_4_ before and after 7000 scans, scan rate 20 mV s^−1^ (not *IR*-corrected). Figure adapted with permission from [3], Copyright 2018 John Wiley and Sons.

Combined inkjet printing and exposure to high intensity pulsed flashlight of thin liquid metal precursor films for the synthesis of metal nanoparticles was first introduced by Lesch [3]. He fabricated platinum NPs (Figure 4A,B) and nanostructures as electrocatalysts on indium tin oxide (ITO)-coated glass slides. ITO was used as electrocatalyst support, one of the materials that is currently in discussion to replace carbon black, which in energy-related electrochemical devices can suffer from corrosion. Thanks to the high standard redox potential, Pt cations can be reduced under relatively mild conditions. Hydrogen hexachloroplatinate (H_2_PtCl_6_) was selected as a metal precursor due to its solubility in aqueous-based inks and the absence of alkali ions that would remain in the printed and light-processed layer in case potassium hexachloroplatinate (K_2_PtCl_6_) would be used. H_2_PtCl_6_ was dissolved in a mixture of water, isopropanol, and 1,2-propanediol in order to fulfill (i) the required physicochemical ink characteristics, such as surface tension and viscosity for printing, as well as good wetting of the substrate surface to create homogeneous and highly resolved patterns, and (ii) the presence of reducing agents to facilitate the reduction of Pt^IV^ to Pt^0^. The photochemical and photothermal processes of Pt^IV^Cl_6_^2−^ generated Pt^0^ on the substrate notably by just one single light flash and, further, only gaseous side products, such as evaporated ink solvents, HCl, CO_2_, and other volatile components.

It was demonstrated by the author that the reduction of Pt^IV^ also took place using just pure water as solvent, thus without the presence of alcohols. It was further confirmed that the metal precursor film absorbed the light and generated the required heat and that it was not the substrate, which absorbed the light for heat generation. To demonstrate this, the author fabricated a pattern of Pt on quartz glass, which was transparent for the wavelengths emitted by the flashlamp. By using spectroscopic analyses (in particular XPS, XRD, and EDS), the complete precursor conversion was confirmed, in particular by the absence of chlorine signals in the printed Pt patterns. ITO-coated glass was selected as the substrate, not only because of its low absorption of the used wavelengths but also because it is conductive, enabling the use of the created Pt/ITO film as an electrode for electrochemical measurements. Electrochemical characterizations demonstrated not only the typical shapes and peaks known for Pt in cyclic voltammograms in acidic solution but also the stability in terms of current for the oxygen reduction reaction over 7000 cycles in 0.1 M HClO_4_ (inset in Figure 4C), where only a minor shift in potential was observed (Figure 4C).

The author made further important observations for Print-Light-Synthesis using a high intensity Xe flashlamp, for instance that the loading of the Pt precursor clearly influenced the process result [3]. Reasonable low loadings of the Pt precursor generated majorly individual nanoparticles. Increasing the loading of the Pt precursor generated larger nano- and microstructures and a high loading generated even conductive Pt patterns on insulators. Therefore, Print-Light-Synthesis is not limited to nanoparticle synthesis but can also be used to fabricate conductive films.

**Figure 5:**
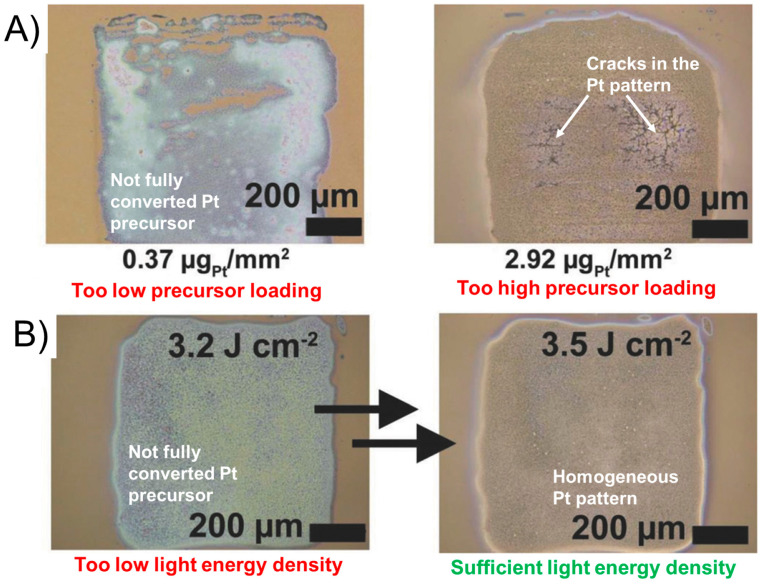
Print-Light-Synthesis with a high intensity Xe flashlamp for the creation of patterns of Pt on indium tin oxide-coated glass slides. Microscopic images demonstrating the effect of precursor loading optimization (**A**) and light energy density limit (**B**). Figure adapted with permission from [3], Copyright 2018 John Wiley and Sons.

Furthermore, a too-low precursor loading on a non-absorbing substrate resulted in incomplete precursor reduction (Figure 5B). This indicated that the process was also based on thermal decomposition as a certain amount of precursor was necessary to generate the required temperature. On the contrary, a too-high precursor loading created forces that were so large that ITO-glass was even cracked (Figure 5A, right). The author further demonstrated the importance of the generated high light intensity, as below a certain intensity the light-induced conversion of the precursor was not complete (Figure 5A, left).

**Figure 6:**
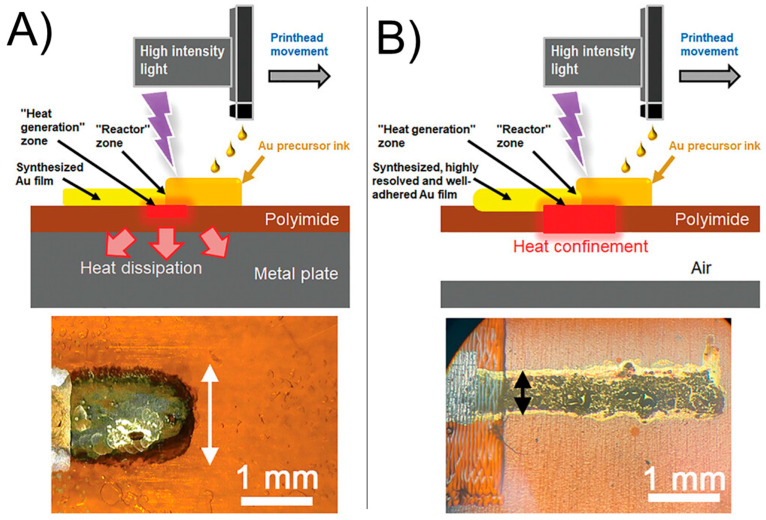
Print-Light-Synthesis with a high intensity Hg arc lamp operated simultaneously to inkjet printing to fabricate thin gold film electrodes. The printing resolution can be increased by controlling the heat distribution in the light absorbing substrate. Placing the substrate on a metal plate transports the heat quickly away (**A**). Introduction of a layer of air, which is a poor heat conductor, increases the local temperature of the substrate limiting pattern widening (**B**). Figure reproduced with permission (open access article distributed under the terms of the Creative Commons CC BY license) from [26].

Apart from creating nanoparticle-decorated electrodes, complete stand-alone thin-film metal electrodes are also of interest for electrochemical applications, as they do not require a conductive support and can be printed directly on an insulating substrate. In a very recent work, Maiorano, Gianvittorio et al. applied Print-Light-Synthesis to generate thin gold films as electrodes on a flexible substrate using a mercury arc lamp [26] (Figure 6). The advantage of using this lamp over the flashlamp is its smaller size and lower costs. A liquid light guide can be used to transport the light from the mercury lamp directly to the printhead, enabling simultaneous printing and photochemical reactions. Inkjet printing was used to generate the thin liquid reaction film with variable gold precursor loading. By using a UV absorbing substrate, in their case polyimide (PI), the substrate transformed light into heat, which was transferred in the above Au precursor ink. Therefore, Print-Light-Synthesis of Au on PI was based on a mixed photochemical and photo-induced thermal reduction process.

The reduction of the Au precursor (i.e., AuCl_4_^−^) was further assisted by the presence of alcohols in the ink. Interestingly, the authors improved the printing resolution (Figure 6A) by introducing a layer of air below the plastic substrate of 150 μm thickness (Figure 6B). This was realized by placing the substrate with a certain distance to the metal printing plate of the printer by introducing a spacer instead of placing the substrate directly on the metal printing plate. In contrary to direct contact between the plastic substrate and the metal plate, air does not quickly dissipate the heat generated in the PI film under UV irradiation, thus creating locally higher temperatures of the substrate. Most likely, this resulted in faster precursor conversion, reducing thus ink spreading.

The Au electrodes were produced either as single nanoparticles on a previously printed conductive graphene layer or as a conductive gold film electrode directly on PI. Alternative substrates were glassy carbon and ITO-coated glass. The Au layers were well adhered and demonstrated clear signals for the electrochemical detection of glucose, 1,4-butanediol, and ascorbic acid.

In fact, obtaining conductive metal films using ink printing and the irradiation of metal precursors is challenging because often inhomogeneously distributed metal nanoparticle aggregates and/or very porous metal layers are generated that do not fulfill the requirements of many applications. In an earlier but much less efficient approach to preparing conductive metal films from metal precursor patterns involving light, Valeton et al. inkjet-printed an Ag precursor ink based on silver neodecanoate, which first had to be activated with UV light and then further chemically reduced by using hydroquinone as the reducing agent [27]. Zope et al. inkjet-printed a solid silver-ligand complex, i.e., μ-oxolato-bis(ethylenediaminesilver(I)), on various substrates and compared thermal reduction on a hot plate and flash light-induced thermal reduction to obtain conductive Ag^0^ patterns [28].

Another research and development focus was the fabrication of Cu patterns. Even though Cu is an unusual bulk electrode material for electrochemistry, it can be interesting as films of individual nanoparticles for electrocatalysis. A flashlight was used to reduce printed Cu precursor patterns (of inorganic as well as organic origin) into conductive Cu^0^ patterns in various ways, often to fabricate Cu traces for electronic circuits [17,29,30,31,32,33]. Song et al. demonstrated the importance of the ink composition in terms of solvents and stabilizers, such as ethyl cellulose and PVP, to obtain reasonable pattern resolution with homogeneous coverage using screen-printing [30]. Copper in general oxidizes quickly in air to copper oxide, which has a reduced conductivity compared to Cu^0^. Instead of thermal processing in an inert or reducing atmosphere, flashlight in the presence of reducing agents in air was used to obtain Cu^0^ patterns free of Cu oxides, demonstrating another time the high potential of flashlight-based nanoparticle synthesis.

Patterning methods alternative to ink- or paste-based printing approaches (which create 2D precursor ink patterns with micrometer lateral pattern resolution) include the localized irradiation of a large liquid phase containing a dissolved metal precursor and either completely covering the target substrate or into which the target substrate is immersed. Compared to Print-Light-Synthesis based on inkjet printing, only the irradiation parameters are controllable and not the localized precursor loading and/or precursor film thickness. Localized irradiation of the precursor film is realized by translating a laser beam following the shape of the desired pattern or by using masks.

The aim of using this approach is generally to fabricate complete conductive metal films and also 3D structures for electronic applications. After light exposure, the non-converted metal precursor must be washed away, representing an additional process step compared to Print-Light-Synthesis involving inkjet printing. Based on the process, the substrate is often heated during metal precursor reduction (to enable or accelerate the process). When incomplete metal precursor reduction takes place, the substrates with printed structures must often be further heated to enhance the electric conductivity by fully reducing the metal precursor films.

Direct laser writing (DLW), for instance, profits from the precise focusing of the irradiated precursor solution to photochemically reduce the metal precursor. Translating the laser focus in space can create 2D patterns and 3D structures as demonstrated, for instance, for Au [34] and Ag [35,36]. Combined with photo-polymerization, the 3D structure is supported and mechanically strengthened by an as-generated polymer, which at high temperature post-treatment processes can be removed, leaving just the metal. However, without such shape-guiding polymers, simple metal pillars can be generated. A surfactant can be added to the precursor solution that acts as a growth inhibitor, enabling the fabrication of nanometric structures [35]. Laser-induced reduction of a Ag precursor on poly(styrene-block-butadiene-block-styrene) also generated elastic patterns of Ag [37].

To increase the rate of photochemical reduction without using a photocatalyst or heat, light irradiation can be combined with electrochemistry [38]. While irradiating a metal precursor solution locally with a laser that is in contact with a conductive substrate, the substrate can be moderately polarized. The applied potential does not provoke direct electrochemical reduction of the metal precursor, which would cause homogeneous metal deposition over the entire polarized metal surface. Local metal patterning is enabled only where the laser irradiates the substrate. The combination of laser irradiation with electrode polarization enables the localized electron transfer from the substrate to the dissolved metal precursor for precursor reduction.

In an alternative approach, masks are used to accelerate the entire process by irradiating the complete area of interest for short periods without the need to translate components (e.g., printhead or light beam). Upon homogeneous irradiation of the entire mask, the mask blocks the light locally, while the non-blocking parts let the light pass for the local photochemical reduction of a metal precursor to M^0^. Reduced pattern resolution could be caused by the diffraction of light, causing blurred instead of sharp pattern edges [39].

To favor the photochemical reduction near the substrate surface for the deposition of metal NPs, the light exposure is often carried out from the bottom through the substrate, which can require that the substrate is transparent to the used wavelengths. Zhang et al. printed Ag and Au patterns photocatalytically using highly resolved UV irradiation of an Ag precursor salt solution that wetted a TiO_2_-coated substrate [40,41]. The loading of Ag was controlled by the process parameters (e.g., light intensity, addition of PVP as stabilizer) as well as the type of substrate, enabling the printing of patterns of Ag gradients that were operated as plasmonic devices. The gold(I) thiourea complex ion Au(SC(NH_2_)_2_)^2+^ was used instead of the tetrachloride gold ion complex AuCl_4_^−^ due to lower standard redox potential (*E*°_[Au(SC(NH_2_)_2_)]_^2+^_/Au_^0^ = +0.38 V vs. SHE instead of *E*°_[AuCl_4_]_^−^_/Au_^0^ = 1.00 V vs. SHE), which required the photocatalyst but enabled higher resolution during photocatalytic metal printing. Zuo et al. created M/MoS_2_ particles (M was Ag and Pt) through photochemical reduction using a laser [42]. Molybdenum is a semiconductor generating electron-hole pairs under irradiation. Pt/MoS_2_ was studied as an electrocatalyst for the hydrogen evolution reaction (HER).

Yang et al. created patterns of citrate-stabilized Ag NPs by way of the photochemical reduction approach using image projection, which means the use of a so-called digital mask and a light projector [43]. The limited electrical conductivity of the Ag patterns compared to bulk Ag had to be improved post-print, by treating the Ag patterns chemically with sodium chloride solution, which removed the organic capping agent that had reduced the inter-Ag NPs conductivity. The image projection approach has recently been further modified as polymer-assisted photochemical deposition [44,45]. Metal nanoparticles were synthesized in solution under UV-irradiation in the presence of a combined capping/reducing agent (citric acid) and immediately captured by a polymer (polyallylamine). Wu et al. reported the beneficial effect of adding anions to increase the conductivity of metal patterns obtained using the light projection approach [46]. Wang et al. printed Ag, Au, and Pd patterns by projecting an image with a blue laser into a solution containing the metal cations [47]. Their work showed that, after photochemical deposition with this approach, loose metal nanoparticles must be washed away from the substrate and additional thermal curing with the laser after printing can be necessary.

### 3.2. Print-Light-Synthesis of Mixed Metal Nanoparticles

**Figure 7:**
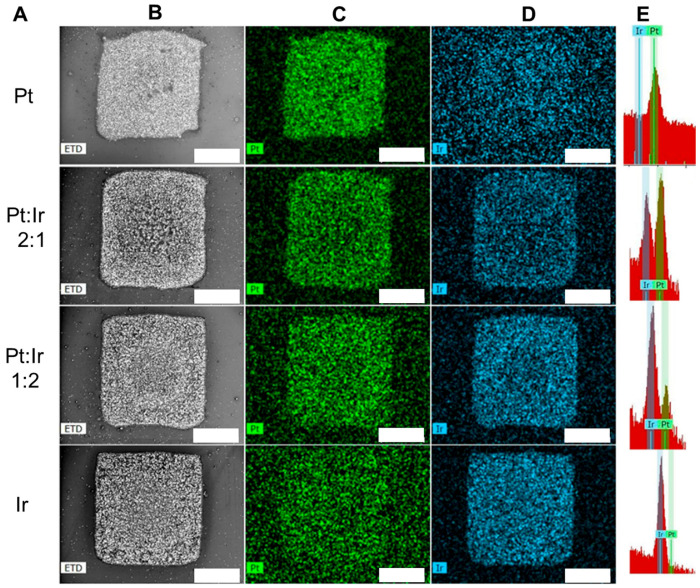
Print-Light-Synthesis of mixed Pt and Ir patterns (3.0 μg total metal precursors weight mm^−2^) on ITO -coated glass slides with different ratios of the two metal precursor inks. Column (**A**): mass ratio between platinum and iridium precursor; (**B**) (greyscale): scanning electron micrographs of the patterns of mixed Pt and Ir after Print-Light-Synthesis; (**C**) (green): EDX maps of Pt; (**D**): EDX maps of Ir; (**D**) (blue): EDX maps of Pt; (**E**): EDX peaks for Ir and Pt. Scale bar 400 μm. Details can be found in the experimental section.

Using inkjet printing for the deposition of metal precursor inks has another technical advantage apart from the precise loading of the precursor that can be achieved on the target substrate. By using different printheads in parallel, each filled with a precursor of a different metal, liquid mixed metal precursor films of well-defined ratios of the metals can be printed. This has also been realized with other ink-based material deposition systems but was usually followed by thermal treatments and not by high intensity light irradiation [48,49]. Parallel inkjet printing of different metal precursor inks in defined volume ratios on top of each other enables their controllable and adjustable mixture just before their exposure to high intensity light. Figure 7 shows an example of mixed Pt and Ir patterns after Print-Light-Synthesis of mixed Pt^IV^Cl_6_^2−^ and Ir^IV^Cl_6_^2−^ inks using a high intensity Xe flash lamp. The total metal precursor loading was 3.0 μg mm^−2^ (experimental details in Appendix A). Electron microscopy and spectroscopic analysis demonstrated the homogenous distribution of both metals in the patterns of 1 mm^2^. Relative EDX intensities further demonstrate the relative ratios of both metal precursors.

Print-Light-Synthesis is also applicable to non-noble metals. This was demonstrated, for instance, by Costa Bassetto et al. who fabricated thin electrode coatings of Ni*_x_*Fe*_y_* nanoparticles that were synthesized on patterns of carbon nanotubes (CNTs) [50]. The ratios of both metal precursors (here, FeCl_2_ and NiCl_2_ dissolved in an aqueous-based ink) were highly controllable by the printing parameters (or alternatively by the ink composition). Due to the low standard redox potentials of both metals (*E*°_Fe_^2+^_/Fe_^0^ = −0.41 V vs. SHE and *E*°_Ni_^2+^_/Ni_^0^ = −0.25 V vs. SHE), direct photochemical reduction of the metal precursors was not efficient with the process parameters and light source used. The process, therefore, required the presence of alcohols as reducing agents.

**Figure 8:**
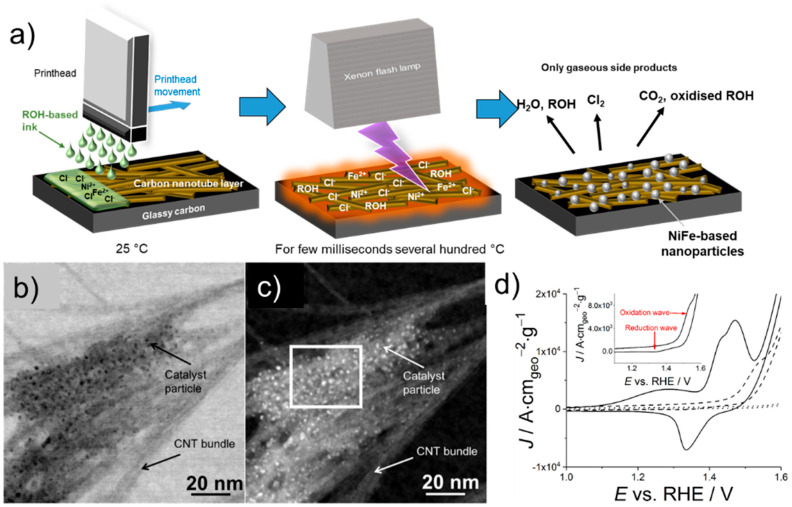
Print-Light-Synthesis of mixed metal nanoparticles supported on carbon nanotubes. (**a**) Schematic representation of the PLS of a NiFe/CNT coated glassy carbon plate. The CNT layer acts as a light-to-heat converter to increase the precursor temperature for enhancing its reduction rate. Bright-field (**b**) and high-angle annular dark-field (**c**) STEM images of Ni_0.45_Fe_0.55_ NPs as formed on CNTs by Print-Light-Synthesis. (**d**) Electrochemical characterization of Ni/CNT/GC (solid line), Ni_0.45_Fe_0.55_/CNT/GC (dashed line and inset), and Fe/CNT/GC (dotted line). Electrolyte 0.1 M KOH, scan rate 50 mV s^−1^, 20th cycle with vertex potentials 0.25 and 1.75 V (an excerpt from the CV between 1.00 V and 1.60 V is plotted). Not *IR*-corrected. Adapted with permission from [50]. Copyright 2019 American Chemical Society.

Furthermore, the authors printed first on the electrode support (i.e., GC) a thin film of CNTs, which acted as a light-to-heat absorber. The efficient absorption of the irradiated light caused a rapid increase of the temperature of the CNTs (Figure 8a). The CNTs then indirectly heated the mixed precursor ink on top and thus accelerated the light-induced reduction of both metal precursors. The synthesized mixed metal nanoparticles of a few nanometers were linked to the CNTs in this way (Figure 8b,c). Based on electrochemical analyses (Figure 8d), in particular for the oxygen evolution reaction (OER) under alkaline conditions, the authors suggested that, in their Print-Light-Synthesis approach, first Fe-based NPs were generated (for instance due to the lower thermal decomposition temperature) and then Ni was incorporated into the Fe-based NPs. Their electrochemical results demonstrated an improvement of the OER characteristics of Fe approaching those of pure Ni. However, Fe is usually incorporated into Ni, thus improving the electrocatalytic activity of Ni. In fact, from this difference it can be stated that light-based synthesis approaches of mixed metals can follow alternative reaction pathways compared to conventional wet syntheses routes.

Examples other than patterning alloys or mixed metals are composites of metals with non-metals. Such composites are generally applied to increase the stability and activity of the metal nanomaterials. In many approaches, the metals are deposited on carbonaceous materials, such as carbon nanotubes and graphene [51]. For instance, by using laser writing Zhou et al. fabricated patterns of a Cu/C composite. Besides an inorganic Cu^II^ precursor, tannic acid was added as carbon precursor to the ink that also contained reducing agents [52]. The authors had the aim of enhancing the resistance of Cu to oxidation.

### 3.3. Oxidative Print-Light-Synthesis

The possibility to create oxidized metal species using Print-Light-Synthesis rather than metals of oxidation state zero is challenging, because the oxidation state of the metal after the process should not be zero and should potentially be even higher than it was in the precursor. One approach was recently realized by Silva et al., who developed an oxidative Print-Light-Synthesis approach to synthesizing Prussian Blue (PB) from ferrous (Fe^II^) salts [53]. Prussian blue and its analogues (PBAs) represent a promising group of materials that is currently considered a potential candidate for the cathode material in alkaline metal ion batteries. In PB, Fe^III^_4_[Fe^II^(CN)_6_]_3_ contains both Fe^2+^ and Fe^3+^. For the Print-Light-Synthesis of PB, a partial oxidation of Fe^II^ to Fe^III^ is, therefore, required. The hexacyanoferrate(II) anion ([Fe(CN)_6_]^4−^) was used as a Prussian Blue precursor.

**Figure 9:**
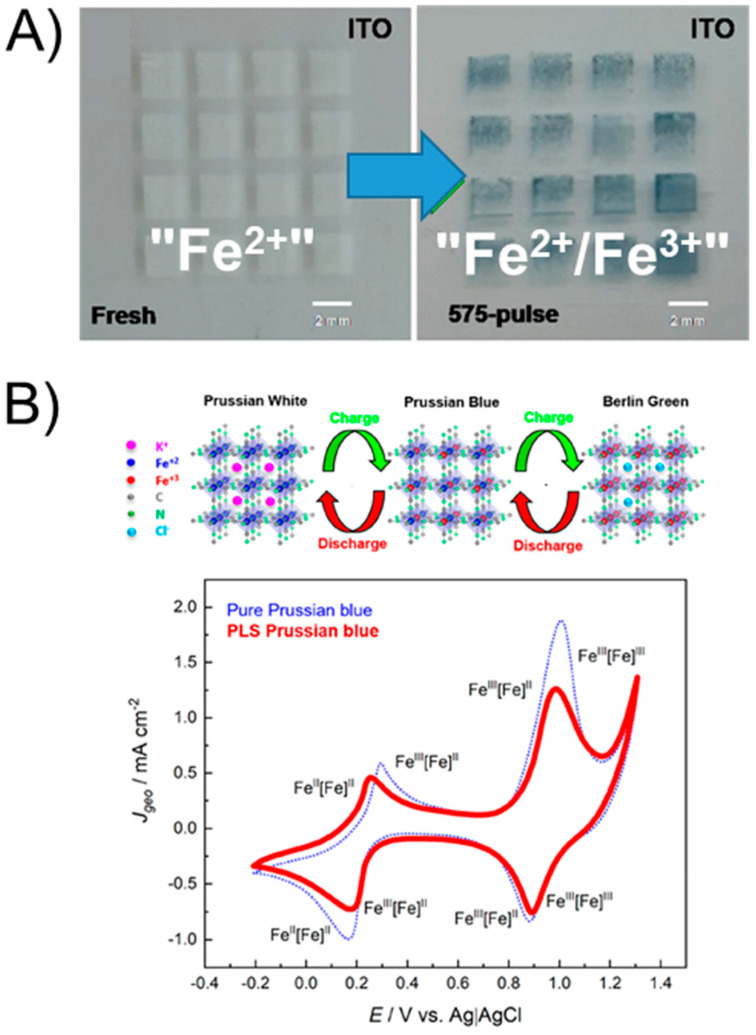
Oxidative Print-Light-Synthesis of patterns of Prussian Blue electrodes. (**A**) Optical photographs directly after inkjet printing of the PB precursor ink and after inkjet printing and flashlight exposure of the as-obtained PB patterns. (**B**) Electrochemical characterization at 10 mV s^−1^ in argon-saturated 1 M KCl + 5 mM HCl (pH = 2.34) of the PB electrode obtained using Print-Light-Synthesis (solid red line) and using conventional PB synthesis (dashed blue line). Adapted with permission from [53]. Copyright 2020 American Chemical Society.

The oxidative PLS was realized by placing the substrate with printed precursor ink patterns immediately into a gas chamber filled with oxygen, creating, therefore, an oxidizing atmosphere to potentially promote higher oxidation states of iron. However, the authors further described the necessary presence of inorganic acids, such as hydrochloric acid and sulfuric acid, to facilitate the redox processes. The authors suggested that the protons might act as electron acceptors, being therefore reduced to H_2_ (or forming HCN) and causing the partial oxidation of the Fe^II^ redox center (the only oxidation state of iron in the precursor salt) to Fe^3+^ (present in the obtained solid film together with Fe^II^ after Print-Light-Synthesis). The result was a blue deposit that the authors characterized electrochemically and spectroscopically as Prussian Blue (Figure 9A).

Notably, under non-acidic conditions the partial oxidation of Fe^II^ to Fe^III^ was indeed not observed, confirming the assumption of the authors that protons must be present as electron acceptors. Electrochemical characterization of the as-obtained PB electrodes in acidic solution demonstrated the typical redox peaks for the oxidations and reductions of the two Fe redox centers in PB (Figure 9B). Kindle et al. used direct laser writing to fabricate patterns of mixed metal oxides. In their approach, solvothermal synthesis for metal oxide preparation was combined with the patterning ability of the laser [54]. Upon thermal decomposition of the precursors, metal oxides were formed. It must be noted, however, that this process worked only with non-noble metals (e.g., Fe, Ni and Cr) that easily generate an oxidized surface, while noble metals (e.g., Au, Pt and Ag) were fully reduced, generating M^0^ patterns instead of patterns of metal oxides. McGee et al. fabricated multimetal (Cr, Fe, Co and Ni) bifunctional electrocatalysts in a similar way for overall water splitting (in particular, for oxygen evolution the oxides are more active than the metals with oxidation state zero) [55]. Castonguay et al. printed mixed metal oxides of Cu, Ni, Zn, and Fe as gas sensing materials [56], and Bae et al. fabricated ZnO and ITO with that approach [57].

## 4. Conclusions

In this review, we presented recent works on Print-Light Synthesis for the fabrication of patterned electrodes and electrode coatings containing metal nanoparticles. Compared to conventional methods that are based on the separate synthesis and patterning of nanoparticles, Print-Light-Synthesis presents the following key advantages: (i) nanoparticle synthesis and patterning of nanoparticles are merged into a one-step process; (ii) thin reaction volumes with highly controllable, very low loadings of metal precursors and ratios of mixed metal precursors are reproducibly prepared using mask-less drop-on-demand ink deposition technologies (we demonstrated in particular the advantage of inkjet printing); (iii) the possibility of operating under ambient conditions (room temperature, atmospheric pressure, presence of oxygen) but also under controlled conditions (e.g., under inert atmosphere) using specific cells; (iv) no post-print processes are required if the process is designed and realized exactly as what is defined herein as Print-Light-Synthesis (i.e., the aimed-for metal nanoparticles are deposited on the substrate while all other ink components escape in the gas phase); (v) the use of industrial technologies, i.e., inkjet printing and high intensity light sources, offers low cost production on industrial scale.

However, in certain cases, there are also difficulties to overcome for Print-Light-Synthesis. The light-induced processes must be rapid and at least as fast as the speed of printing. Otherwise, post-processing will be required to convert the remaining precursor and/or to wash away the non-fully-converted precursor. Print-Light-Synthesis is straightforward for noble metals with high standard redox potentials but requires some additional components in the process when metals of low standard redox potential shall be converted. Examples include the addition of alcohols as reducing agents and the use of light-to-heat absorbers. Despite these advantages and the promising future applications of Print-Light-Synthesis, the approach requires deeper investigations in terms of the design and realization of advanced nanomaterials. As has been demonstrated for mixed Ni and Fe nanoparticles, the synthesis result of materials from the metal precursors when using irradiation can differ from thermal and chemical approaches. Therefore, mechanistic studies should be deepened. In addition, the control of specific shapes of nanoparticles shall be further addressed. However, it can be foreseen that, in the near future, the range of methods—not limited to Print-Light-Synthesis—for the sustainable and large-scale production of thin films of nanomaterials will expand and advance due to the imminent need to reduce material consumption and to link laboratory and industrial scale fabrications.

## Data Availability

The data that support the findings of this study are available from the corresponding author upon reasonable request.

## Appendix A. Experimental Details

The results presented in Figure 7 were obtained as described in the following.

Chloroplatinic acid hexahydrate (H_2_PtCl_6_·x6H_2_O; 38–40% Pt; 99.9% Pt; Strem Chemicals), hydrogen hexachloroiridate(IV) hydrate (H_2_Cl_6_Ir·xH_2_O, 36.0–44.0% Ir, Sigma-Aldrich), isopropanol, and 1,2-propanediol (Sigma) were of analytical grade and used as received. DI water (18.2 MΩ cm) was produced by using a Milli-Q water purification system with a Q-POD element. ITO (In_2_O_3_/SnO_2_)-coated 1 mm thick glass slides were used as substrates and purchased from Sigma.

For inkjet printing of mixed metal precursor patterns, an X-Serie CeraPrinter (Ceradrop, Limoges, France) was employed using two piezoelectric-based cartridges DMC-11610 (Fujifilm Dimatix, Lebanon, NH, USA) with 16 individually addressable nozzles and 10 pL nominal droplet volume. One cartridge contained the Pt precursor ink and the other cartridge contained the Ir precursor ink. The ratio between the two metal precursors on the substrate was controlled by the printing parameters. The ITO-coated glass substrates were pre-treated in isopropanol in order to ensure a surface free energy higher than the surface tension of the metal precursor inks for improved wetting. All inkjet printing parameters, including the jetting frequency and droplet ejection pulse by piezoelectric actuation were adjusted for optimum printing resolution. The substrate plate was heated to 54 °C. Flashlight treatment of the printed mixed metal precursor patterns was performed with a PulseForge 1300 system (Novacentrix, Austin, USA) that was fully integrated into the CeraPrinter enabling Print-Light-Synthesis to be carried out. A single pulse of 330 μs duration was applied. The energy was adjusted by controlling the lamp driver charging voltage, i.e., 765 V. The patterns were then analyzed by using a Fei Teneo scanning electron microscope with EDX spectroscopy (XFlash Silicon drift detector).
